# Polyhydroxy butyrate biosynthesis by *Azotobacter chroococcum* MTCC 3858 through groundnut shell as lignocellulosic feedstock using resource surface methodology

**DOI:** 10.1038/s41598-022-15672-y

**Published:** 2023-07-03

**Authors:** Kasilingam Nagajothi, A. G. Murugesan

**Affiliations:** 1 Sri Paramakalyani Centre of Excellence in Environmental Sciences, Manonmaniam Sundaranar University, Alwarkurichi, 627412 Tamil Nadu India; 2 Dept. of Microbiology, K.R. College of Arts and Science, Kovilpatti, 628503 Tamil Nadu India

**Keywords:** Biotechnology, Microbiology, Environmental sciences

## Abstract

This work appraises the prospect of utilising groundnut shell hydrolysate as a feedstock used for PHB biosynthesis by *Azotobacter chroococcum* MTCC 3853 under SMF conditions. Sugar reduction: untreated and pretreated 20% H_2_SO_4_ (39.46 g/l and 62.96 g/l, respectively), untreated and enzymatic hydrolysis (142.35 mg/g and 568.94 mg/g). The RSM-CCD optimization method was used to generate augment PHB biosynthesis from groundnut shell hydrolysate (30 g/l), ammonium sulphate (1.5 g/l), ammonium chloride (1.5 g/l), peptone (1.5 g/l), pH 7, 30 °C, and a 48 h incubation time. The most convincing factors (*p* < 0.0001), coefficient R^2^ values of biomass 0.9110 and PHB yield 0.9261, PHB production, highest biomass (17.23 g/l), PHB Yield(11.46 g/l), and 66.51 (wt% DCW) values were recorded. The control (untreated GN) PHB yield value of 2.86 g/l increased up to fourfold in pretreated GN. TGA results in a melting range in the peak perceived at 270.55 °C and a DSC peak range of 172.17 °C, correspondingly. According to the results, it furnishes an efficient agricultural waste executive approach by diminishing the production expenditure. It reinforces the production of PHB, thereby shrinking our reliance on fossil fuel-based plastics.

## Introduction

The worldwide need for and enhancement in the manufacture of fuel-based polymers has substantially improved in the last few years. Synthetic fuels are derived from petroleum-derived polymers such as polyurethane, polypropylene, polytetrafluoroethylene, and their malleability, stability, and resistance^1^. In 2019, the worldwide production of plastics reached 367 million tonnes^2^. Scrapping the usage of plastic waste causes environmental pollution and health hazards. Alternative sources used for degradable biopolymers can be synthesised from various renewable resources (starch, cellulose, and lignin-based polymers) and microbial biopolymers (polyhydroxyalkanoates [PHAs])^3^.

Polyhydroxybutyrate (PHB) is a monomer of polyhydroxyalkanoates (PHA) that is biosynthesized, amassed in intracellular lipid granules, and produced by plenty of microorganisms. polyhydroxyalkanoates (PHB) are biodegradable, brittle, renewable, low elastic, thermoplastic, biocompatible, environmentally safe, resilient, low molecular weight (5–10 × 105), non-toxic and hydrophobic in nature, and they have physical, thermal and mechanical properties similar to synthetic polymers^3,4^. PHB biopolymer has numerous applications in medicine, agriculture, automobiles, packaging, and cosmetic industries^5^.

*Bacillus* sp., *Azotobacter* sp*., Cupriavidus* sp., and *Pseudomonas* sp. were among the dynamic microbes that produced PHB biopolymer. (i) Acetoacetyl-CoA was formed when the enzyme-ketothiolase (phaA) reacted with acetyl-CoA. (ii) The enzyme acetoacetyl-CoA reductase (phaB) catalyses the acetoacetyl-CoA reduction reaction, which produces (R)-3-hydroxybutyryl-CoA. PHB synthase (phaC) catalyses the conversion of (R)-3-hydroxybutyryl-CoA to PHB^6^. PHB is produced from carbon rich and limited in nitrogen and phosphorus nutrients, which are essential for microbial growth^7^. It makes the biosynthesis of PHB through Bacillus spp and the biodynthesis of co-polymers more valuable^8–10^. PHA is produced and accumulated by a variety of microorganisms, accounting for up to 90% of cell dry weight^4,11^.

In pilot scale PHB production medium cost is more expensive than alternative substrate of numerous bio-waste like carbohydrate (lignocellulose), lipids, and proteins^12^. However, boosting the bioprocess is economically beneficial because it is used for both microbial growth and producing a dynamic byproduct. It may be more profitable to least production costs and maintained the waste management^13^. Biowastes are low-cost, renewable, easily available, and biodegradable materials derived from agro-industrial waste resources such as pea shell slurry^9,14^, coconut coir^15,16^,coffee waste^17^, paper mills^18^ and glycerol^19^. In this study focused in groundnut shell, the worldwide production of groundnuts increased up to 50.7 million tonnes in 2022^20^. PHB production from lignocellulosic waste residues has been treated with acid, enzymatic hydrolysis. The massive complexity and heavy association of the contents of lignocellulose bring about a great structure and hindrance for PHB biosynthesis^21,22^.

This study, PHB production from pretreated GN wastes used for fermentation by Azotobacter chroococcum MTCC 3858 strain has an excess of carbon sources and stores PHB granules in the cytoplasm^23^. PHB production produced the yield of biomass, PHB, and weight percent of DCW and was optimised through RSM CCD design and characteristic analysis.

## Result

### Acid and enzymatic hydrolysis of GN

This study focused on groundnut shell biowaste substrates used to biosynthesize PHB by *A. chroococcum* MTCC 3858. The untreated GN (%w/w) composition was as follows: cellulose 39.46 ± 0.3%; hemicellulose 13.47 ± 0.5%; lignin 24.18 ± 0.5%; ash 4.63 ± 0.02%; moisture, 6.12 ± 0.5%. After pretreatment, the GN consists of 65.96 ± 0.5% cellulose, 7.14 ± 0.2% hemicellulose, and 14.32 ± 0.2% lignin (Table S1). Acid hydrolysis (H_2_SO_4_) at different concentrations was used for pre-treating groundnut shells. 20% H_2_SO_4_ was found to be the best concentration to produce the highest reducing sugar yield of 62.96 ± 0.5 g/l when used at 120 °C for 1 h of incubation. The highest reducing sugar was found to be 46.84 ± 0.01 g/l after 1 h of incubation at 90 °C, and the highest reducing sugar was 38.92 ± 0.2 g/l after 1 h of incubation at 60 °C (Table 1).

**Table 1:** Pretreated acid hydrolysis of groundnut shell.

Incubated Period	Concentration of H_2_SO_4_				
(1 h)	1%	2%	5%	10%	20%
60 °C	2.65 ± 0.2	8.26 ± 0.3	15.26 ± 0.5	22.67 ± 0.1	38.92 ± 0.2
90 °C	4.86 ± 0.7	11.67 ± 0.5	22.67 ± 0.05	29.32 ± 0.02	46.84 ± 0.01
120 °C	6.74 ± 0.3	13.89 ± 0.02	27.29 ± 0.75	43.48 ± 0.2	62.96 ± 0.5

Each value is calculated as the mean ± SD of the triple times analysis.

The enzymatic hydrolysis of pretreated GN was performed using cellulase from *Aspergillus nigar* (50 FPU/g) for 96 h. After hydrolysis increased the results pretreated GN (78.62%) was 4 times higher than the untreated GN (19.52%). Overall, reduced sugar (cellulose) yield from both untreated GN (9.84 ± 0.01 g/l), and pretreated GN (46.81 ± 0.01 g/l) was achieved (Table 2). An untreated GN yields 142.35 mg/g of sugar, while a pretreated GN yields 568.94 mg/g of sugar.

**Table 2:** Enzymatic hydrolysis of groundnut shell.

	12 h	24 h	36 h	48 h	60 h	72 h	84 h	96 h
Untreated GN (g/l)	3.16 ± 0.1	4.97 ± 0.5	5.09 ± 0.15	7.46 ± 0.25	9.84 ± 0.5	9.84 ± 0.01	9.84 ± 0.01	9.84 ± 0.01
Pretreated GN (g/l)	14.87 ± 0.4	22.56 ± 0.15	28.42 ± 0.2	36.72 ± 0.1	42.68 ± 0.3	44.32 ± 0.02	44.79 ± 0.02	46.81 ± 0.01

Each value is calculated as the mean ± SD of the triple times analysis.

### RSM-CCD optimization process

The optimization results for increasing PHB production are shown in Table 3. RSM CCD design in each independent variable was coded at three levels of low, middle, and high, and the four factors. The model data given to the variables are coded values and the actual value levels. The CCD design ran thirty experiments, and regression on the empirical data was successful. The equations of polynomials were analysed and converted into empirical and presumed values.

**Table 3:** RSM-CCD empirical and predict values of dependent factors for PHB production by *A. chroococcum* from groundnut shell hydrolysate.

Run	Factor 1	Factor 2	Factor 3	Factor 4	Response 1	Response 2	Response 3
	A:Groundnut shell	B:Amm. Sulfate	C:Ammonium Chloride	D:Peptone	Biomass Yield	PHB Yield	% PHB
	g/l	g/l	g/l	g/l	g/l	g/l	g/l
1	30	1.5	1.5	1.5	17.23	11.46	66.51
2	20	1	1	1	13.78	7.12	51.66
3	20	1	2	2	13.24	7.05	53.24
4	30	1.5	1.5	1.5	17.23	11.46	66.51
5	40	2	1	2	15.26	9.12	59.76
6	20	1	1	2	13.14	6.85	52.13
7	20	2	2	1	13.24	7.05	53.24
8	20	2	2	2	14.29	7.42	51.92
9	10	1.5	1.5	1.5	9.34	4.52	48.39
10	40	1	2	2	15.26	9.12	59.76
11	30	1.5	1.5	1.5	17.23	11.46	66.51
12	40	2	1	1	15.26	9.12	59.76
13	40	2	2	2	15.18	9.23	60.8
14	30	1.5	1.5	0.5	17.15	10.18	59.35
15	40	1	2	1	14.95	9.02	60.33
16	30	1.5	1.5	1.5	17.23	11.46	66.51
17	20	2	1	1	12.98	6.52	50.23
18	40	1	1	1	14.23	8.79	61.77
19	40	1	1	2	14.38	8.85	61.54
20	30	1.5	1.5	2.5	17.19	10.34	60.15
21	30	1.5	2.5	1.5	17.19	10.34	60.15
22	30	0.5	1.5	1.5	17.15	10.18	59.35
23	30	1.5	1.5	1.5	17.23	11.46	66.51
24	20	2	1	2	13.24	7.05	53.24
25	30	1.5	1.5	1.5	17.23	11.46	66.51
26	30	1.5	0.5	1.5	17.15	10.18	59.35
27	30	2.5	1.5	1.5	17.19	10.34	60.15
28	20	1	2	1	13.14	6.85	52.13
29	40	2	2	1	15.26	9.12	59.76
30	30	1.5	1.5	1.5	17.23	11.46	66.51

The ANOVA results noted the value of the probability model F > 0.0001 and revealed that the quadratic model was the most significant. The F-values for biomass of 10.96 (Table 4), and PHB Yield of 13.43 (Table 5), suggest the model is significant. The highest coefficient of determination was revealed, with R2 values of biomass of 0.9110 and a PHB yield of 0.9261. The predicted R squared values for biomass were 0.2901, 0.3954 for PHB Yield, while the adjusted R-squared values were 0.8279, 0.8571 for PHB Yield. Adequate precision response results when the ratio of greater than 4 is desirable. The ratio of biomass yield is 16.0908, the PHB yield is 16.4732, which indicates an adequate signal and the coefficient value of > 10 is tolerable. The correlation between empirical and presumed values was good. The model was validated successfully.

**Table 4:** ANOVA for the quadratic model for PHB production from groundnut shell hydrolysate feedstock: biomass.

Source	Sum of squares	Coefficient estimate	df	Mean square	F-value	*p* value
Model	105.19		14	7.51	10.96	< 0.0001^S^
**Intercept**		17.23				
A-Groundnut shell	6.26	0.6045	1	6.26	9.14	0.0086
B-Amm. sulfate	0.2970	0.1113	1	0.2970	0.4334	0.5203
C-Ammonium chloride	0.2340	0.0988	1	0.2340	0.3415	0.5676
D-Peptone	0.0630	0.0513	1	0.0630	0.0920	0.7658
AB	0.1785	0.1056	1	0.1785	0.2605	0.6172
AC	0.0352	0.0469	1	0.0352	0.0513	0.8239
AD	0.0095	− 0.0244	1	0.0095	0.0139	0.9078
BC	0.0018	0.0106	1	0.0018	0.0026	0.9597
BD	0.1073	0.0819	1	0.1073	0.1565	0.6980
CD	0.1620	0.1006	1	0.1620	0.2364	0.6338
A^2^	69.05	− 2.05	1	69.05	100.76	< 0.0001
B^2^	1.18	− 0.2061	1	1.18	1.72	0.2092
C^2^	1.18	− 0.2061	1	1.18	1.72	0.2092
D^2^	1.18	− 0.2061	1	1.18	1.72	0.2092
**Residual**	10.28		15	0.6853		
Lack of fit	10.28		9	1.14		
Pure error	0.0000		6	0.0000		
Cor total	115.47		29			
Std. dev.	0.8278			R^2^	0.9110	
Mean	15.33			Adjusted R^2^	0.8279	
C.V. %	5.40			Predicted R^2^	0.2901	
PRESS	81.97			Adeq precision	16.0908	

Significant^S^.

**Table 5:** ANOVA for the quadratic model for PHB production from groundnut shell hydrolysate feedstock: **PHB Yield.**

Source	Sum of squares	Coefficient estimate	df	Mean square	F-value	*p* value
Model	96.60		14	6.90	13.43	< 0.0001^S^
**Intercept**		11.46				
A-Groundnut shell	12.46	0.8525	1	12.46	24.25	0.0002
B-Amm. Sulfate	0.0704	0.0542	1	0.0704	0.1370	0.7164
C-Ammonium Chloride	0.1291	0.0733	1	0.1291	0.2512	0.6235
D-Peptone	0.0840	0.0592	1	0.0840	0.1635	0.6917
AB	0.0256	0.0400	1	0.0256	0.0498	0.8264
AC	0.0030	− 0.0137	1	0.0030	0.0059	0.9399
AD	0.0196	− 0.0350	1	0.0196	0.0381	0.8478
BC	0.0210	0.0363	1	0.0210	0.0409	0.8424
BD	0.0529	0.0575	1	0.0529	0.1029	0.7528
CD	0.0132	0.0288	1	0.0132	0.0257	0.8747
A^2^	45.23	− 1.66	1	45.23	88.03	< 0.0001
B^2^	6.30	− 0.4763	1	6.30	12.26	0.0032
C^2^	6.30	− 0.4762	1	6.30	12.26	0.0032
D^2^	6.30	− 0.4763	1	6.30	12.26	0.0032
**Residual**	7.71		15	0.5139		
Lack of fit	7.71		9	0.8564		
Pure error	0.0000		6	0.0000		
Cor total	104.31		29			
Std. dev.	0.7168			R^2^	0.9261	
Mean	9.15			Adjusted R^2^	0.8571	
C.V. %	7.83			Predicted R^2^	0.3954	
PRESS	63.06			Adeq precision	16.4732	

Significant^S^.

**Figure 1:**
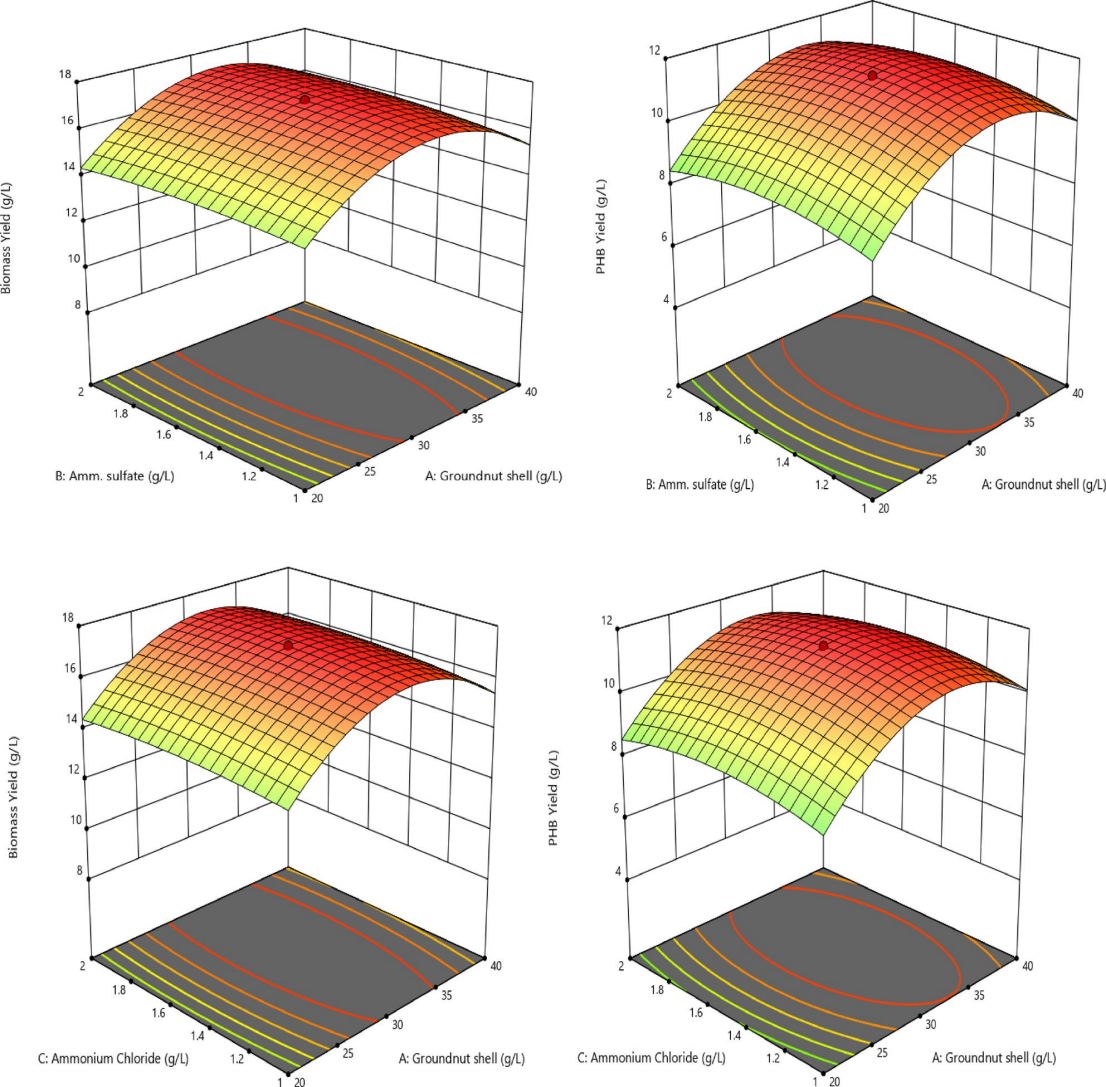

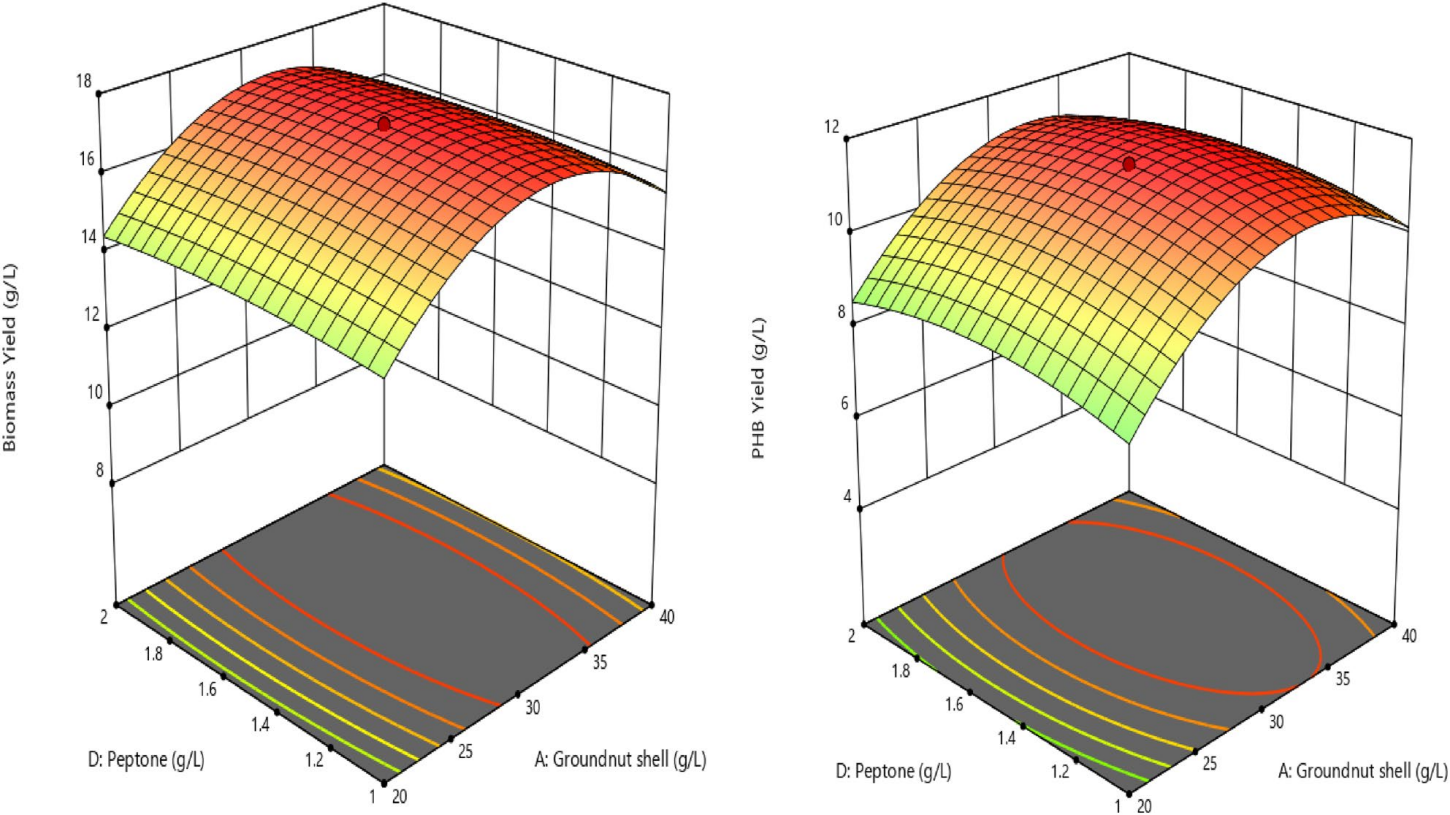
RSM Optimization of PHB Production from groundnut shell hydrolysate by *Azotobacter chroococcum*—Biomass and PHB Yield.

In the present work, the regression equation was observed to have the maximum PHB production. GN hydrolysate (30 g/l), ammonium sulphate (1.5 g/l), ammonium chloride (1.5 g/l), peptone (1.5 g/l), pH 7, temperature 30 °C, incubation time 48 h, biomass yield (CDW) 17.23 ± 0.12 g/l, PHB Yield 11.46 ± 0.5 g/l and 66.51% PHB accumulation, with 95% conformity with the assumption were the factors. Untreated GN biomass yield 8.37 ± 0.2 g/l, PHB Yield 2.86 ± 0.15 g/l and 34.16% PHB accumulation and PHB yield fourfold increased in pretreated GN produced (Table S2). The PHB production and optimization processes were repeated three times. The model was validated and optimum results for maximum PHB production and their interactions, contour plots, and RSM 3D curves were plotted in Fig. 1.

### Characterization of PHB by *A. chroococcum*

#### FT-IR analysis

**Figure 2:**
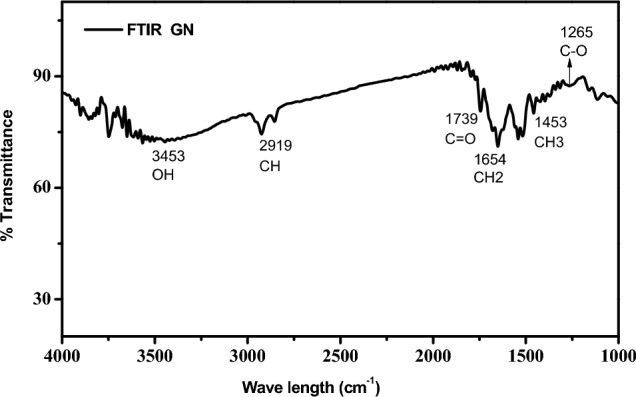
Fourier-transform infrared spectroscopy (FTIR) analysis of PHB from groundnut shell hydrolysate by *Azotobacter chroococcum*.

In this present work, purified PHB was analyzed for functional groups through FTIR spectroscopy results. The peak value is noted in Fig. 2. It expressed the various functional groups, such as (Alcohol, alkane, polyester, aldehyde, alkyl, aryl ether) the wave number 3453.12 cm^−1^ corresponding to the alcohol, polyester functional group (O–H), and the band at 2919.44 cm^−1^ indicates alkane functional group (CH). The C=O group, band represents the well-known stretching and the peak at 1739.26 cm^−1^ aldehyde, ester functional group. The bands at 1654.36 (CH2) and 1453.46 cm^−1^ express the CH3 bend (aldehyde, alkane functional group) and another stretch represents that stretching C–O group. All these peaks of PHB were noted in 1265.12 (C–O stretching) (alkyl, aryl ether functional group).

#### NMR analysis

**Figure 3:**
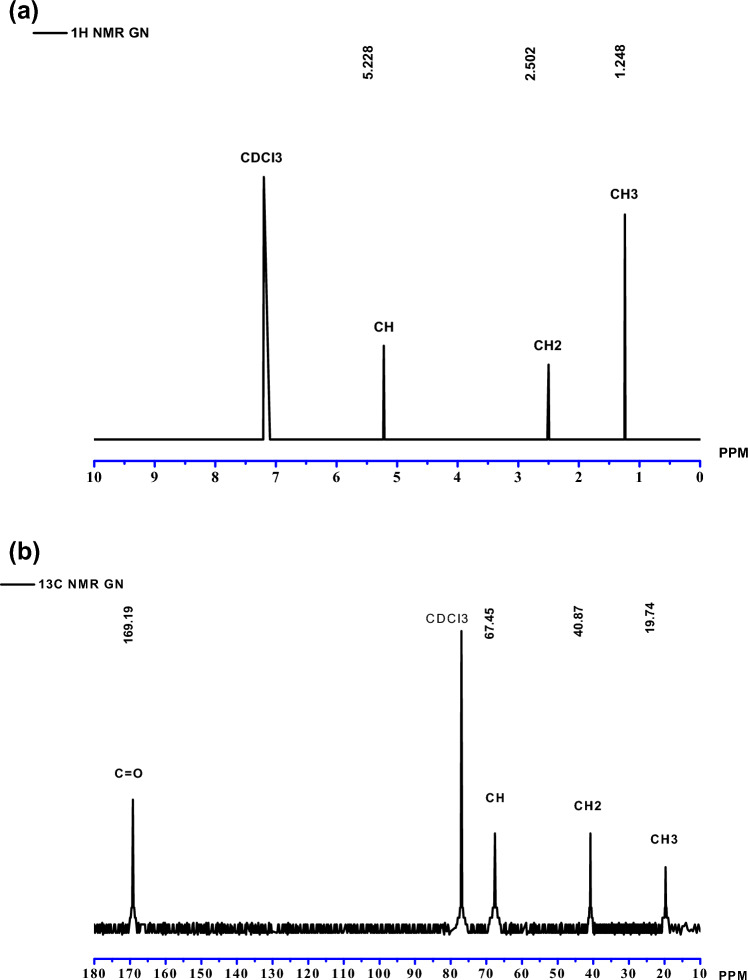
(**a**) ^1^H NMR analysis of PHB extracted by *Azotobacter chroococcum* from GN hydrolysa. (**b**) ^13^C NMR analysis of PHB extracted from groundnut shell hydrolysate by *Azotobacter chroococcum*.

In this present work, the production and purified form of PHB from GNhydrolysate residues were analyzed by using ^1^H NMR spectroscopy graph plotted in Fig. 3a. The NMR spectrum (400 MHz, CDCl3) value of PHB agreed with various carbon atoms. It clarified with dominant peaks at 1.248 ppm attained from the absorption of the CH3 group; another peak at 2.502 ppm from the absorption of the CH2 group, and a 5.228 ppm peak for methane (CH) groups. ^13^C NMR spectrum analysis (400 MHz, CDCl3) yielded peak results of 19.74 ppm due to CH3 (methyl group), 40.87 ppm due to CH2 (methylene), 67.45 ppm due to CH (methane), and 169.19 ppm due to C=O (carbonyl) functional group (Fig. 3b).

#### Thermogravimetric (TGA) and differential scanning calorimetry (DSC) analysis

**Figure 4:**
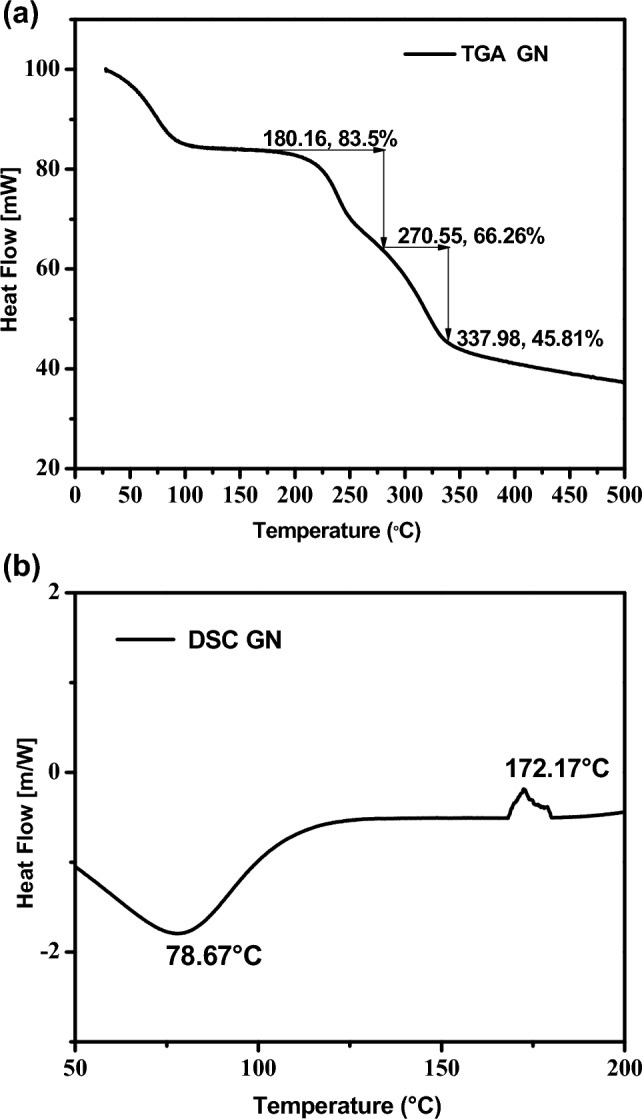
(**a**) Thermogravimetric analysis (TGA) analysis of PHB extracted from groundnut shell hydrolysate by *Azotobacter chroococcum*. (**b**) Differential scanning calorimetry (DSC) analysis PHB is extracted from groundnut shell hydrolysate by *Azotobacter chroococcum.*

In the present study, thermogravimetric analysis (TGA) of the PHB produced from GN hydrolysate demonstrated in Fig. 4a. The TGA peak obtained for PHB degradation of the sample noted a 30–500 °C temperature range under an inert atmosphere (N2) for the weight loss of PHB. As TGA achieves maximum thermal degradation at a temperature of 250 °C, it could be combined with ester component breakage for PHB biopolymer by an eradication reaction. Melting temperatures for the first stage of degradation were 180.16 °C, 270.55 °C for the second stage of degradation, and 337.98 °C for the third stage of degradation, with mass percentages of 83.5%, 66.26%, and 45.81%, respectively. The PHB sample was exposed to DSC analysis to detect peak values representing the melting temperature (Tm) range of 300 °C at 10 °C/min heat flow. The results of DSC analysis of the melting temperature (*T*m)for PHB sample peak values at 172.17 °C, − 0.219 m/W/mg, and glass transition (*T*g) were attained to be 5.2 °C, respectively (Fig. 4b).

## Discussion

Recently, researchers have investigated the PHB of biosynthesis from lignocellulose residues as a substrate under laboratory conditions^3^. This study, which produced PHB strain A. chroococcum in a pre-screened process, was carried out by the Sudan Black method^24^. In the acid hydrolysis process, 15% of the peanut shell was added with 1% sulfuric acid, and after 6 h of incubation, the reducing sugar amount increased from 36.4 to 71.68%^4,25^. Teff straw (1 g) mixed with 10 ml of 4% sulfuric acid at 120 °C for 60 min to produced 41.8 wt% of the highest reducing sugars^26^. Similarly, 72 h of enzymatic pretreatment in peanut shell with cellulose (10 FPU g1) at 190 °C and 60 bar CO_2_ pressure produced an 80.7% glucose yield^27^. Wheat bran mixed with β-glucosidase of Aspergillus niger (50 CBU/g) to reduce the sugar yield of untreated and pretreated wheat bran (159 and 629.1 mg/g) respectively^28^. Enzymatic pretreatment of the yield of reducing sugars to obtained in 72.67 mg/g of untreated rice husk and 266.5 mg/g of pretreated rice husk^29^.

In present study RSM CCD was optimized to biosynthesis of PHB fourfold increased, highest biomass (17.23 g/l), PHB Yield (11.46 g/l), 66.51 (wt% DCW) and R2 values biomass 0.9110 and PHB yield 0.9261 were recorded. Similarly production of PHB from peanut shell by *Bacillus* sp to produced highest PHB (945–1205 mg^−1^) and Yield (55–65% w⁄w)^29^. Coconut coir as an agricultural substrate produced PHB content at 64.7% (w/w) and an improved 3.7-fold in PHB yields^16^. Maximum production of PHA from cardboard industry waste water by *Enterococcus* sp. NAP11 produced 79.27% and *Brevundimonas* sp. NAC1 produced 77.63% and PHA concentration of 5.236 g/l and 4.042 g/l^30^. Produced biosynthesis of PHB from pulp waste, biosynthesizing 61.7% of DCW reported that R2 values (0.9837 and 0.9735)^31^. Similar results RSM Optimization process to improve the production of PHB from rice bran, PHB yield attained the value of F = 14.389 and *p* < 0.005 for the strong significant model and R^2^ = 0.963^32^. PHB biosynthesised from banana peel extract by marine seaweeds (identified as *Pichia kudriavzevii* VIT-NN02) produced biomass yield from 40.00 ± 0.1 to 79.68 ± 0.2% and RSM results R^2^ values of biomass (0.9904) and PHB content (0.9926)^33^.

In the FTIR section of the study, the PHB from the selected candidate strain expressed the various functional groups obtained, such as carbonyl group, alcohol, alkane, polyester, aldehyde, alkyl aryl ether), wave length of (3453.12, 2919.44, 1739.26, 1654.36, 1453.46, 1265.12 cm^−1^). Similarly FT-IR analysis obtained the PHB characterization results, 2950.56 absorption peaks represent CH2 groups and another peak value of 2556.60 represents CH3 groups, absorption bands at 1750.8 (C=O group), 1350.60 (C–O group), and 1260.6 (C–O stretching)^34^. Similarly another peak at 1720 cm^−1^ represents an ester group (C=O stretch), absorption bands were obtained at 2976.1 and 2933.4 cm^−1^ (aliphatic CH3, CH2 groups) respectively^35^. Similarly FT-IR spectral analysis of PHB correlated with the earlier report, suggesting the isolated compound as PHB peak values 1721 (C=O group), 1380 (C–O group), and 1277 (CH stretching), 2975,2933 (CH2, CH3), 3443 (OH)^36^.

^1^H NMR spectrum analysis, in this study, the extracted PHB sample expressed the peak value (1.24, 2.502, and 5.228 ppm) representing the methyl, methylene, and methane groups. Similarly, the peak values were noted at 1.23 ppm for methyl group (CH3) absorption, 2.5 ppm for methylene (CH2O–COOH) absorption, and 5.2 ppm for methane (HC=CH) groups^37^. ^1^H NMR spectrum analysis results shows 5.25 (methane), 2.56 (methylene), and 1.58 (methyl) ppm^34^. Another study found a similar peak assigned to protons in the methane (5.26 ppm), methylene (2.7–2.4 ppm) and methyl (1.28 ppm) groups of PHB^38^.

In the 13C NMR spectrum analysis in this study, the extracted PHB sample expressed the peak values of 19.74 (methyl group), 40.87 (methylene), 67.45 (methane), and 169.19 ppm due to the carbonyl functional group. Similarly, 13C NMR spectrum results show 19.58 ppm (CH3), 40.72 ppm (CH2), 67.56 ppm (CH), and 169.11 ppm (C=O) respectively^38^. Similarly, 13C NMR results in extracted PHB were 19.770 ppm (CH3), 40.810 ppm (CH2), 67.618 ppm (CH), and 169.134 ppm (C=O)^39^. Another work noted similar results with a peak allocated to carbonyl (159.77–158.16 ppm), PHB carbon resonances of methane (67.5 ppm), methylene (37.60 ppm), and methyl (14.87 ppm)^40^.

In the TGA section of this study, the PHB from the selected candidate strain thermal degradation peak value was 270.55 °C and completely decomposed at 337.98 °C. Previous studies have discovered that the PHB TGA result, the PHB degrade temperature of 288 °C, the ester bond breaks, and the PHB sample completely degrades at 320°C^36^. Previous research has shown that these results are comparable to PHB-synthesized endothermic peak values at 178°C^17^. TGA analysis of the peak melting temperature of PHB at 260 °C was obtained^41^. In the DSC section of this study, the PHB from the chosen candidate strain had a melting temperature range of 172.17 °C and a Tg of 5.2. Similarly, the extracted PHB in DSC analysis of the melting temperature (Tm) was 171.8 and the glass transition temperature (Tg) was 4.03 °C, respectively^36^. DSC analysis extracted the PHB melting point peak value at 171 °C^41^. Another study found similar results in DSC analysis of the melting temperature range and PHB Tm peak at 175 °C (water hyacinth-hexose rich (or enzymatic) hydrolysate), 173 °C (water hyacinth–pentose rich (or acid) hydrolysate), 177 °C (P. hysterophorus—hexose rich (or enzymatic) hydrolysate), 173 °C (P. hysterophorus—pentose rich (or acid) hydrolysate)^42^.

## Conclusion

In this present study, it could be assumed that enhanced production of PHB biosynthesis from cheap, easily available groundnut shell hydrolysate lignocellulosic residues. PHB increased fourfold in 30 g/l of GN hydrolysate by *A. chroococcum* produces the maximum results biomass 17.23 ± 0.5 g/l, PHB Yield 11.46 ± 0.02 g/l and 66.51% of PHB. It confirmed that the characterization of PHB was carried out by methods such as FT-IR, NMR, TGA, and DSC and that various parameters were used for fermentation and RSM optimization. It was decided to control environmental pollution and health hazards while decreasing the non-degradable manufacture of polymers. RSM design Groundnut shell hydrolysate successfully well- established as an encouraging statistical tool to enhance PHB yield from GNhydrolysate by *A. chroococcum* MTCC 3853, which can be utilized to pilot scale up the production of PHB bio-polymers of industrial interest.

## Materials and methods

The Microbial Type Culture Collection, Chandigarh, India, purchased the strain *A.chroococcum* MTCC 3853 used for the fermentation study. Cultivated it in nutrient broth by incubating it at 30 °C and storing the culture in glycerol (1:1, v: v) at − 40 °C.

### Pretreatment of GN

The lignocellulosic raw material GN hydrolysate was collected from the regional agricultural field of Kovilpatti, Tamilnadu, India. GN are washed in tap water several times to remove impurities like sand and dried in an oven at 60 °C, followed by grinding it properly to a fine powder (a thickness of 0.5 mm) and storing it in sterile containers at room temperature for future experiments.

### Acid and enzymatic hydrolysis of GN hydrolysate

Lignocellulose substrate (1 g) treated with acid (H_2_SO_4_ various concentrations of 1%, 2%, 5%, 10%, and 20%) oven at different temperatures 60, 90, 121 °C for 60 min using the NREL/TP-510-42618 standard methodologyfollowed^43^. Enzyme hydrolysis followed by the NREL/TP-5100-63351 standard protocol, 10% of GN in citrate buffer (50 mM), 50 FPU/g cellulase, pH 5, incubated at 60 °C and 200 rpm for 72 h^44,45^. After regular intervals estimate the reducing sugars both the initial substrate and the enzyme hydrolysate substrate by Dinitrosalicylic acid method is used to. The number of enzymes that produce 1 µmol of reduced sugars per minute is estimated as the cellulase unit. All the experiments were performed in triplicates after centrifugation to collect clear GN hydrolysate, which was used for fermentation studies.

### RSM CCD optimization of polyhydroxybutyrate biosynthesis from GNhydrolysate

The production of PHB under SMF conditions as per the methodology followed^32,45^ made minor modification of mineral salt medium to contain the following ingredients in g/l: 1.0 g Na_2_HPO_4_.2H_2_O, 1.5 g of KH_2_PO_4_, 0.5 g of NH_4_Cl, 0.5 g of MgSO_4_·7H_2_O, 0.05 g CaCl_2_·2H_2_O, 1.2 mg of Fe (III) NH_4_-citrate with pre-treated GN hydrolysate hydrolysate, pH 7, was inoculated with 1 × 10^–5^ cells/ml of *A. chroococcum* culture.

The biosynthesis of PHB optimize by RSM using CCD designs according to the methodology^46,47^. The RSM CCD software tool (version 13, Stat-Ease, Inc.) was used to analyse at α-value of ± 2 levels of the variables to optimize. Four factors, such as A- GNhydrolysate (20, 30 and 40 g/l), B- Ammonium sulphate (1.0, 1.5, 2.0 g/l), C-Ammonium chloride (1.0, 1.5, 2.0 g/l), D-Peptone (1.0, 1.5, 2.0 g/l), pH 7, 30 °C, and incubated for 48 h, shaker at 150 rpm. Each attempt was carried out three times, and the results were calculated as the values by using the consecutive equation.

### Extraction and quantification of polyhydroxybutyrate from GNhydrolysate

Harvested PHB cells were extracted and quantified in accordance with the slightly modified methodology using NaOCl and H_2_SO_4_^46,48^. After centrifugation, purified PHB was collected, and this procedure was performed in triplicates at 4 °C to store the sample for further studies.

### Characterization of biosynthesized PHB from GNhydrolysate

#### Fourier transform-infrared spectroscopy (FTIR) analysis

The extracted PHB functional groups were decisive through FTIR spectroscopic analysis as per the methodology^34–36^. 2 mg of extracted PHB sample was mixed with 200 mg of potassium bromide (KBr) and the spectrum peak was estimated at 500 to 4000 cm^−1^ using the Shimadzu model (RF6000).

#### Nuclear magnetic resonance spectroscopic analysis

The characterization of PHB was carried out through 1H & 13C NMR analysis^38–40^. The 1H & 13 C NMR spectrum analysis was seized at 400 MHz using the Bruker Advance Model. The PHB sample was dissolved in 10 mg/ml^−1^ of deuteron chloroform (CDCl3) solvent used to interpret it.

#### Thermogravimetric and differential scanning calorimetry of biosynthesized PHB from GN hydrolysis

The thermal stability of the extracted PHB was further characterised through TG and DSC analysis^36,41,42^. 5 mg of sample and a temperature range of 30–500 °C, with a boiling rate of 10 °C/min in an N gas atmosphere (nitrogen flow rate of 40 ml/min). TG and DSC were determined by Netzsch, model STA 449F3. Melting temperature (Tm), glass transition (Tg), crystallization (Tc) were determined. X_c_ = ΔHf/ω. H_f_^o^ × 100 where heat of fusion of the samples (ΔHf), the heat of fusion (H_f_^o^) for 100% crystallized PHB (146 J/g) and the mass fraction (ω) for the PHB biopolymer.

## Supplementary Information

Supplementary Tables.

Supplementary Information.

## Data Availability

All data generated or analysed during this study are included as supplementary information file.
