# Supplementary material for: Polyhydroxy butyrate biosynthesis by Azotobacter chroococcum MTCC 3858 through groundnut shell as lignocellulosic feedstock using resource surface methodology
Source: Sci Rep. 2023 Jul 3;13:10743. doi: 10.1038/s41598-022-15672-y (PMC10318099; doi:10.1038/s41598-022-15672-y)
Supplement: Supplementary file 1 — Supplementary Tables. [file 41598_2022_15672_MOESM1_ESM.docx]

**Table S1. Chemical composition of Untreated and Pretreated Groundnut shell**

| Component | Untreated Groundnut shell  (% Dry wt) | Pretreated Groundnut shell  (% Dry wt) |
| --- | --- | --- |
| Cellulose | 39.46±0.3 | 65.96±0.5 |
| Hemicellulose | 13.47±0.5 | 7.14±0.2 |
| Lignin | 24.18±0.5 | 14.32±0.2 |
| Ash | 4.63±0.02 | 5.38±0.5 |
| Moisture | 6.12±0.5 | 7.29±0.1 |

Each value is calculated as the Mean ± SD of the triple times analysis.

**Table S2. Untreated groundnut shell**

| **Untreated GN (g/L)** | **Biomass** | | | | **PHB Yield** | | | |
| --- | --- | --- | --- | --- | --- | --- | --- | --- |
|  | **Test 1** | **Test 2** | **Test 3** | **Average** | **Test 1** | **Test 2** | **Test 3** | **Average** |
|  | 8.57±0.23 | 8.41±0.19 | 8.21±0.22 | 8.37±0.2 | 3.34±0.23 | 2.96±0.27 | 3.24±0.19 | 2.86±0.15 |

Each value is calculated as the Mean ± SD of the triple times analysis
