# Supplementary material for: Polyhydroxy butyrate biosynthesis by Azotobacter chroococcum MTCC 3858 through groundnut shell as lignocellulosic feedstock using resource surface methodology
Source: Sci Rep. 2023 Jul 3;13:10743. doi: 10.1038/s41598-022-15672-y (PMC10318099; doi:10.1038/s41598-022-15672-y)
Supplement: Supplementary file 2 — Supplementary Information. [file 41598_2022_15672_MOESM2_ESM.docx]

**Raw Data’s**

**Table 1 Chemical composition of Untreated and Pretreated Groundnut shell**

| **Component** | **Percentage of Dry weight** | | | | | | | |
| --- | --- | --- | --- | --- | --- | --- | --- | --- |
|  | **Untreated Groundnut shell** | | | | **Pretreated Groundnut shell** | | | |
|  |  | | | |  | | | |
|  | **Test 1** | **Test 2** | **Test 3** | **Average Value** | **Test 1** | **Test 2** | **Test 3** | **Average Value** |
| Cellulose | 38.97±0.2 | 39.52±0.3 | 39.89±0.4 | 39.46±0.3 | 65.98±0.51 | 64.42±0.46 | 67.48±0.52 | 65.96±0.5 |
| Hemicellulose | 13.56±0.5 | 13.18±0.3 | 13.67±0.7 | 13.47±0.5 | 7.09±0.2 | 7.22±0.7 | 7.11±0.7 | 7.14±0.2 |
| Lignin | 23.98±0.47 | 24.29±0.55 | 24.27±0.48 | 24.18±0.5 | 14.28±0.23 | 14.36±0.24 | 14.32±0.19 | 14.32±0.2 |
| Ash | 4.58±0.018 | 4.69±0.021 | 4.62±0.019 | 4.63±0.02 | 5.51±0.54 | 5.29±0.39 | 5.34±0.57 | 5.38±0.5 |
| Moisture | 6.18±0.48 | 6.01±0.53 | 6.17±0.49 | 6.12±0.5 | 7.19±0.18 | 7.42±0.06 | 7.28±0.12 | 7.29±0.1 |

Each value is calculated as the Mean ± SD of the triple times analysis.

**Table 2 Acid hydrolysis (Raw data) (Page No :4)**

| Incubation Period | H_2_SO_4_ (Acid treatment) | Test 1 | Test 2 | Test 3 | Average |
| --- | --- | --- | --- | --- | --- |
| 60⁰C | 1% | 2.53±0.14 | 2.94±0.1 | 2.48±0.16 | 2.65±0.2 |
|  | 2% | 8.41±0.29 | 8.22±0.24 | 8.15±0.23 | 8.26±0.3 |
|  | 5% | 15.17±0.46 | 14.98±0.49 | 15.63±0.45 | 15.26±0.5 |
|  | 10% | 22.68±0.12 | 22.49±0.06 | 22.84±0.18 | 22.67±0.1 |
|  | 20% | 38.76±0.22 | 40.12±0.21 | 37.88±0.17 | 38.92±0.2 |
| 90⁰C | 1% | 4.93±0.67 | 4.68±0.72 | 4.97±0.71 | 4.86±0.7 |
|  | 2% | 12.76±0.49 | 11.27±0.42 | 10.98±0.41 | 11.67±0.5 |
|  | 5% | 22.84±0.04 | 22.43±0.06 | 22.74±0.05 | 22.67±0.05 |
|  | 10% | 29.12±0.019 | 30.06±0.016 | 28.78±0.015 | 29.32±0.02 |
|  | 20% | 47.76±0.02 | 46.79±0.01 | 45.97±0.01 | 46.84±0.01 |
| 120⁰C | 1% | 6.82±0.26 | 6.59±0.3 | 6.81±0.28 | 6.74±0.3 |
|  | 2% | 13.92±0.02 | 13.81±0.01 | 13.94±0.03 | 13.89±0.02 |
|  | 5% | 27.52±0.68 | 27.13±0.74 | 27.22±0.67 | 27.29±0.75 |
|  | 10% | 42.89±0.11 | 43.51±0.18 | 44.04±0.13 | 43.48±0.2 |
|  | 20% | 63.02±0.52 | 62.79±0.45 | 63.07±0.47 | 62.96±0.5 |

Each value is calculated as the Mean ± SD of the triple times analysis.

**Table 3. Enzymatic hydrolysis (Raw data) (Page No :4)**

| Sources | Experiments | 12hr | 24hr | 36hr | 48hr | 60hr | 72hr | 84hr | 96hr |
| --- | --- | --- | --- | --- | --- | --- | --- | --- | --- |
| Untreated GN (g/L) | Test 1 | 3.08±0.14 | 4.83±0.51 | 5.12±0.13 | 7.52±0.25 | 9.79±0.56 | 9.81±0.01 | 10.12±0.01 | 9.96±0.02 |
|  | Test 2 | 3.42±0.12 | 5.16±0.48 | 4.98±0.14 | 7.37±0.18 | 10.13±0.49 | 9.65±0.02 | 9.92±0.01 | 9.91±0.01 |
|  | Test 3 | 2.98±0.08 | 4.92±0.49 | 5.17±0.12 | 7.49±0.26 | 9.60±0.55 | 10.06±0.01 | 9.48±0.02 | 9.65±0.01 |
|  | Average | 3.16±0.1 | 4.97±0.5 | 5.09±0.15 | 7.46±0.25 | 9.84±0.5 | 9.84±0.01 | 9.84±0.01 | 9.84±0.01 |
| Pretreated GN (g/L) | Test 1 | 14.73±0.39 | 22.49±0.15 | 28.39±0.18 | 36.91±0.12 | 43.35±0.26 | 44.28±0.03 | 44.87±0.02 | 47.73±0.01 |
|  | Test 2 | 15.03±0.41 | 22.31±0.25 | 28.36±0.24 | 35.96±0.19 | 41.98±0.2 | 44.22±0.01 | 44.81±0.01 | 46.41±0.02 |
|  | Test 3 | 14.85±0.38 | 22.88±0.13 | 28.51±0.22 | 37.29±0.07 | 42.71±0.24 | 44.46±0.02 | 44.69±0.03 | 46.29±0.01 |
|  | Average | 14.87±0.4 | 22.56±0.15 | 28.42±0.2 | 36.72±0.1 | 42.68±0.3 | 44.32±0.02 | 44.79±0.02 | 46.81±0.01 |

Each value is calculated as the Mean ± SD of the triple times analysis

**Table 4: Optimization of different factors using triple time analysis (Raw data) (Page No :5)**

| Factors | | | | Biomass | | | | PHB yield | | | |
| --- | --- | --- | --- | --- | --- | --- | --- | --- | --- | --- | --- |
| A | **B** | **C** | **D** | **Test 1** | **Test 2** | **Test 3** | **Average** | **Test 1** | **Test 2** | **Test 3** | **Average** |
| 30 | 1.5 | 1.5 | 1.5 | 17.20±0.14 | 17.56±0.11 | 16.95±0.09 | 17.23±0.12 | 11.59±0.44 | 9.46±0.48 | 13.33±0.46 | 11.46±0.5 |
| 20 | 1 | 1 | 1 | 13.90±0.12 | 13.70±0.18 | 13.74±0.14 | 13.78±0.2 | 7.49±0.07 | 6.89±0.08 | 6.98±0.06 | 7.12±0.07 |
| 20 | 1 | 2 | 2 | 13.31±0.12 | 12.96±0.14 | 13.45±0.09 | 13.24±0.14 | 6.95±0.27 | 7.12±0.31 | 7.08±0.28 | 7.05±0.3 |
| 40 | 2 | 1 | 2 | 14.21±0.48 | 17.71±0.39 | 13.86±0.41 | 15.26±0.46 | 9.24±0.42 | 9.15±0.53 | 8.97±0.45 | 9.12±0.5 |
| 20 | 1 | 1 | 2 | 13.19±0.15 | 12.97±0.18 | 13.26±0.13 | 13.14±0.2 | 6.42±0.41 | 6.79±0.44 | 7.34±0.39 | 6.85±0.42 |
| 20 | 2 | 2 | 2 | 13.43±0.12 | 16.47±0.13 | 12.97±0.16 | 14.29±0.15 | 7.28±0.07 | 7.54±0.05 | 7.44±0.06 | 7.42±0.04 |
| 10 | 1.5 | 1.5 | 1.5 | 7.64±0.03 | 8.42±0.03 | 11.96±0.04 | 9.34±0.1 | 5.12±0.06 | 3.55±0.02 | 4.89±0.05 | 4.52±0.03 |
| 40 | 1 | 2 | 2 | 16.79±0.19 | 14.23±0.23 | 14.76±0.15 | 15.26±0.27 | 8.96±0.04 | 9.32±0.06 | 9.08±0.02 | 9.12±0.04 |
| 40 | 2 | 2 | 2 | 15.01±0.52 | 15.57±0.58 | 14.96±0.5 | 15.18±0.6 | 9.57±0.13 | 8.74±0.14 | 9.38±0.11 | 9.23±0.16 |
| 30 | 1.5 | 1.5 | 0.5 | 17.6±0.28 | 16.87±0.3 | 16.98±0.25 | 17.15±0.23 | 10.35±0.07 | 9.87±0.03 | 10.32±0.04 | 10.18±0.08 |
| 40 | 1 | 2 | 1 | 14.79±0.15 | 14.84±0.28 | 15.22±0.11 | 14.95±0.32 | 9.18±0.09 | 8.93±0.06 | 8.95±0.11 | 9.02±0.04 |
| 20 | 2 | 1 | 1 | 12.71±0.19 | 13.75±0.16 | 12.48±0.17 | 12.98±0.18 | 5.97±0.04 | 6.74±0.07 | 6.85±0.06 | 6.52±0.05 |
| 40 | 1 | 1 | 1 | 14.31±0.08 | 14.4±0.13 | 13.98±0.17 | 14.23±0.04 | 8.43±0.48 | 8.98±0.39 | 8.96±0.45 | 8.79±0.42 |
| 40 | 1 | 1 | 2 | 14.83±0.58 | 14.02±0.61 | 14.29±0.57 | 14.38±0.62 | 8.79±0.39 | 8.84±0.45 | 8.92±0.42 | 8.85±0.36 |
| 30 | 1.5 | 1.5 | 2.5 | 17.28±0.04 | 17.25±0.05 | 17.04±0.07 | 17.19±0.02 | 10.45±0.56 | 9.86±0.49 | 10.71±0.54 | 10.34±0.47 |
| 30 | 1.5 | 2.5 | 1.5 | 17.22±0.02 | 17.10±0.04 | 17.25±0.03 | 17.19±0.03 | 10.39±0.23 | 9.89±0.28 | 10.74±0.33 | 10.34±0.18 |
| 30 | 0.5 | 1.5 | 1.5 | 16.98±0.49 | 17.34±0.48 | 17.13±0.41 | 17.15±0.56 | 10.23±0.08 | 9.97±0.09 | 10.34±0.13 | 10.18±0.3 |

Factors A: Groundnut shell, Factor B: Ammonium sulfate, Factor C: Ammonium chloride, Factor D: Peptone
